# The Social–Psychological Consequences of COVID-19: An Integrative Review and Research Agenda

**DOI:** 10.3390/ijerph23020179

**Published:** 2026-01-30

**Authors:** Jasper Van Assche

**Affiliations:** 1 Center for Social & Cultural Psychology (CESCUP), Université Libre de Bruxelles, 1050 Brussels, Belgium; jasper.van.assche@ulb.be; 2 Optentia Research Unit, North-West University, Vanderbijlpark 1900, South Africa

**Keywords:** COVID-19, social–psychological consequences, mental health, social identity, moral psychology, resilience, prosocial behavior, crisis preparedness

## Abstract

The COVID-19 pandemic has revealed profound social–psychological vulnerabilities and strengths across societies worldwide. Beyond its immediate health implications, the pandemic has triggered a wave of mental health issues, disrupted social cohesion, and challenged community resilience. This paper synthesizes the current literature, critically discusses five recent studies as part of the Special Issue “Mental Health Consequences of COVID-19: The Role of Social Determinants”, and articulates an agenda for future research within a social–psychological framework. Moving beyond mere negative effects such as anxiety, this review highlights the role of resilience, prosocial behavior, (digital) mental health interventions, and community social capital. Correspondingly, I advocate for interdisciplinary efforts to enhance awareness, preparedness, and adaptive capacity during health crises, emphasizing the need for a clearer focus on vulnerable social groups. In sum, recognizing the evolving global landscape, this work underscores the urgency of integrating psychological insights into public health policies to build resilient societies capable of confronting future pandemics and health emergencies.

## 1. The Impact of COVID-19 on Social–Psychological Well-Being

The outbreak of COVID-19 has precipitated a global health crisis unprecedented in modern history. As nations grapple with containing the virus, the societal implications have become increasingly evident. While the initial focus was on understanding the biological behaviors of the SARS-CoV-2 virus (i.e., within the family of coronaviruses, the specific virus causing the COVID-19 disease), a significant body of research has emerged highlighting the deep social–psychological impacts of the pandemic [1]. Research demonstrates that the pandemic has exacerbated existing mental health issues, precipitated new ones, and compromised social cohesion [2]. The pervasive threat of infection, coupled with enforcements of quarantine and social distancing, has amplified feelings of loneliness, anxiety, depression, and stress among diverse populations [3]. The social–economic consequences, including unemployment and financial insecurity, have further compounded mental health challenges, particularly among vulnerable groups [4].

Moreover, the pandemic has unveiled social–psychological disparities rooted in gender, age, socioeconomic status, and cultural factors. For instance, women often experience higher levels of pandemic-related psychological distress, partly due to increased caregiving responsibilities and social pressures [5]. Younger individuals report elevated stress and anxiety levels driven by uncertainty and disruption of social and educational activities [6]. These differences underscore the importance of understanding social determinants of mental health within a pandemic context. In order to accomplish this, this Editorial first reviews three key theoretical frameworks that will serve as handgrips when integrating specific research findings.

## 2. Theoretical Perspectives on Social–Psychological Consequences

Theories of social identity, moral psychology, and community resilience provide crucial frameworks for understanding these phenomena. Social identity theory proposes that individuals derive part of their self-concept from membership in social groups, which becomes particularly salient during collective threats. Health crises can pose such a threat and can undermine group cohesion by increasing intergroup anxiety, stigma, or “us–them” distinctions, yet they can also strengthen ingroup solidarity and mobilize collective efficacy when responses are coordinated and inclusive [7,8]. Recent extensions, such as the social identity approach to health, demonstrate that strong, positive group identities are associated with better mental health and cooperative behavior during crises.

Moral psychology offers complementary insights by explaining how moral emotions and intuitions shape prosocial action. Empathy, fairness-based reasoning, and moral obligation can motivate behaviors such as helping, volunteering, or adhering to protective measures—behaviors that buffer individuals and communities against negative mental health outcomes [9,10]. Research on moral foundations theory [11] further shows that different moral concerns (e.g., harm/care, loyalty, authority) can either promote solidarity or exacerbate divisions depending on how leaders frame public health messages. Thus, moral psychology illuminates why some populations respond with heightened cooperation, while others react with conflict or moralized blame.

Finally, resilience frameworks examine the psychological and social resources that enable individuals and communities to withstand, adapt to, and recover from adversity. Classic work conceptualizes resilience not simply as a personality trait but as a dynamic process involving coping strategies, meaning making, and access to supportive social networks [12,13]. Community resilience research highlights the role of collective narratives, social capital, and institutional trust in sustaining well-being during prolonged crises. These frameworks help explain why some communities rebound quickly or even experience “post-traumatic growth”, while others face long-term psychological strain. In the next section, I will delve further into particular consequences of the coronavirus pandemic.

## 3. Social–Psychological Sequelae of COVID-19

Extensive reviews confirm that the pandemic has precipitated a surge in mental health disorders globally. A systematic review by Xiong and colleagues [2] estimates the prevalence of anxiety and depression increased by up to 45% during the pandemic. Mental health disparities are linked to social determinants, including economic inequality, gender, and cultural context. Such findings resonate with the social determinants of health models that posit health outcomes are shaped by broad social factors [14]. Still, more research is needed in order to structurally compare gaps in mental health related to COVID, for instance, regarding the role of gender.

Research also shows that the pandemic triggered specific emotional responses, such as fear, anger, and helplessness, that influence behavioral and psychological outcomes [15]. Fear of infection and uncertainty about the future contribute to sustained stress, which can impair immune functioning and increase susceptibility to illness [16]. Yet, it remains unclear whether these processes occur to the same extent within each individual. An interesting lacuna to tackle would be to uncover whether the level of COVID-19 anxiety differs between clinical treatment groups and non-clinical control subjects.

Besides the clear negative personal and social costs of the coronavirus and governmental policies to reduce its spread, some positive consequences have been noted. An emerging body of research emphasizes the importance of prosocial behaviors (i.e., altruism, helping strangers, adherence to health guidelines) as mechanisms to foster social cohesion and resilience during crises [17]. Such behavior promotes collective efficacy, mitigates adverse mental health effects, and enhances community adaptability [18]. Empirical evidence indicates that communities with high social capital demonstrate greater resilience, better psychological outcomes, and more effective burden-sharing during a pandemic [19]. As such, a focus on positive outcomes could be considered a third important lacuna to address.

## 4. Advancements in Understanding and Gaps

This section aims to synthesize emerging research, critically analyze five recent articles provided in the Special Issue “Mental Health Consequences of COVID-19: The Role of Social Determinants” (https://www.mdpi.com/journal/ijerph/special_issues/mental_health_consequences_covid19_role_social_determinants, accessed on 16 December 2025), and articulate future research directions emphasizing social–psychological frameworks.

First, Hwang and Shin [20] examined the gender gap in mental health during the pandemic’s first year in South Korea, focusing on labor market experiences. Their quantitative study used a large sample of over 3000 respondents from the “COVID-19-Related Survey for Employees”. Second, Domosławska-Żylińska, Krysińska-Pisarek and Włodarczyk [21] determined the gender-specific level of pandemic fatigue and concerns expressed in relation to the pandemic in Poland. They too launched a large quantitative survey including over 1000 respondents during the fourth wave of the COVID-19 pandemic. Third, Grajek and colleagues [22], again in Poland, estimated the level of COVID-19 anxiety among a very unique sample of oncology patients, making group comparisons between 300 treatment and 300 control participants. Fourth, turning away from (only) negative outcomes, Thomson, Spiro, Williamon and Chatterjee [23] investigated psychological well-being, social connectedness and loneliness in a smaller-scale and larger-scale survey (encompassing over 300 and over 3600 UK citizens, respectively). Finally, Wider, Xian Lim, Shing Wong, Kit Chan and Maidin [24] offer a critical literature review with general insights into prosocial behavior during the COVID-19 pandemic, and specific recommendations for the Malaysian context.

### 4.1. Lacuna 1: The Role of Gender

Hwang and Shin [20] show that women experienced significantly poorer mental health than men during the first year of the COVID-19 pandemic in South Korea and that this gap was strongly linked to gendered labor-market experiences. Women were more likely to face job loss, income reduction, and restricted access to remote work, factors that explained a substantial portion of their elevated levels of depression and anxiety. Particularly vulnerable were women in their thirties, who shouldered both heightened employment insecurity and intensified household and childcare responsibilities. Overall, this study suggests that the gender gap in mental health during the pandemic emerged not from gender alone, but from the intersection of economic disruption, traditional gender roles, and unequal divisions of labor inside and outside the home.

To extend this line of research, several promising follow-up designs can be pursued. A longitudinal panel tracking the same individuals across different phases of the pandemic and into the recovery would allow researchers to identify causal pathways and understand whether gendered mental health disparities widen, diminish, or persist over time. Additional survey measures capturing unpaid care work, work-family conflict, and time-use patterns would help clarify the role of domestic burdens that were not fully accounted for in the original study. Mixed-methods approaches, including in-depth interviews with women in highly affected age groups, could further illuminate the mechanisms through which economic stress, caregiving expectations, and social norms shape mental health. Finally, cross-national or intersectional comparative studies (examining variations across job types, household structures, socioeconomic groups, and different welfare regimes) would help reveal how context shapes vulnerability and resilience and inform more targeted gender-sensitive policies.

Domosławska-Żylińska and colleagues [21] investigated gender differences in pandemic fatigue and COVID-19-related concerns during the fourth wave of the pandemic. Their findings indicate that women reported significantly higher levels of emotional exhaustion, worry, and perceived burden than men. Women were more likely to express concerns about health risks, long-term social and economic consequences, and the well-being of family members, whereas men tended to report lower overall fatigue and fewer pandemic-related fears. These results align with broader international evidence suggesting that women have shouldered a disproportionate psychological burden throughout the pandemic, shaped by gendered stressors (see also [20]). Incorporating additional stressors such as caregiving load, employment instability, domestic role expectations, and access to social support would help to further clarify which factors most strongly predict gender gaps in pandemic fatigue. Furthermore, diary studies, could illuminate how men and women experience pandemic stressors differently in their daily lives.

### 4.2. Lacuna 2: A Closer Look at Vulnerable Populations

Grajek and colleagues [22] explored the level of COVID-19 anxiety among a uniquely vulnerable population: oncology patients undergoing treatment in Poland. They found that oncology patients reported substantially higher levels of pandemic-related fear, worry, and psychological distress: over 80% of oncology patients showed clinically relevant anxiety symptoms compared to just over half of the control group. Cancer patients were also more likely to fear losing their lives to the virus and experienced more frequent sleep disturbances linked to heightened anxiety. These findings highlight how pre-existing health vulnerabilities interacted with pandemic-related threats to produce disproportionately high anxiety levels among individuals already coping with chronic and life-threatening illnesses. Crucially, these elevated anxiety levels should be understood not only in terms of diminished social support but also as the result of a cumulative life stressor burden, particularly within the social domain. According to cumulative stress and allostatic load frameworks, the accumulation of stressors across life domains can overwhelm adaptive capacities and heighten psychological vulnerability [25,26]. From this perspective, pandemic-related anxiety among oncology patients reflects not a single stressor but the convergence of ongoing illness-related stress with acute social and environmental disruptions.

To deepen this line of inquiry, future studies could monitor how COVID-19-related anxiety develops over the course of cancer treatment and survivorship, particularly as pandemic conditions evolve. Including additional psychological variables (such as perceived treatment disruption, trust in medical institutions, risk for distress, social support, and coping strategies) would help to refine the mechanisms driving heightened anxiety in this group. Qualitative interviews could provide richer insight into the lived experiences of oncology patients navigating dual health threats, revealing how fears of infection intersect with cancer-related stress. Finally, cross-country comparisons or analyses of healthcare system factors would help identify structural conditions that either exacerbate or buffer pandemic-related anxiety in medically vulnerable populations.

### 4.3. Lacuna 3: A Focus on Positive Outcomes

Turning toward more positive psychosocial outcomes, Thomson and colleagues [23] examined psychological well-being, social connectedness, and loneliness during lockdowns. Their findings show that cultural, artistic, and environmental engagement (e.g., participating in creative activities, listening to music, attending online arts events, or spending time in nature) significantly reduced feelings of loneliness and strengthened social connectedness during periods of enforced isolation. Participation in the arts and nature exposure functioned as widely accessible mental health resources, helping individuals cope with social restrictions and maintain a sense of belonging and emotional well-being. These results suggest that not all pandemic experiences were uniformly negative. Indeed, engagement with culture and the natural environment provide meaningful avenues for psychological resilience. Building on these compelling findings, follow-up studies could address issues of sustainability, accessibility and inequality: there may not be a lasting impact, and not all communities may have equal opportunities to participate in cultural activities or access green spaces, and marginalized groups may face structural barriers that limit these benefits. Investigating these disparities through intersectional analyses, community-based research, or policy evaluations would help to ensure that the mental health advantages of cultural and environmental engagement can be equitably distributed.

Finally, the analysis of Wider and colleagues [24] discusses how prosocial actions (such as volunteering, providing support to vulnerable groups, and complying with public health measures) can bolster collective resilience and buffer against the social and psychological strains of the pandemic. By synthesizing evidence across disciplines, the authors identify key obstacles to prosociality, including structural inequalities, competing social norms, and variations in trust. They further propose targeted interventions grounded in social identity processes, moral elevation, and community engagement, arguing that strengthening shared identity and social trust can cultivate prosocial motivations at scale. As a result, the review positions prosocial behavior not merely as an individual moral choice but as a critical social resource in times of crisis.

Despite its broad contributions, the study [24] also underscores the need for stronger empirical validation to establish causal pathways between specific prosocial behaviors and improved mental health outcomes. Experimental or quasi-experimental designs would allow researchers to test whether interventions that promote volunteering, solidarity, or mutual aid directly enhance psychological well-being and under what conditions. Moreover, because prosocial motivations and expressions vary across cultural contexts, future research must adopt culturally sensitive designs that consider local social norms, historical experiences, and community structures. Although the review highlights promising intervention successes in Malaysia, their effectiveness in other cultural settings remains to be tested, as noted in recent large-scale replication efforts [27,28]. In line with Van Assche’s “universality without uniformity” principle [29], it is probable that prosociality-based interventions generate broadly positive outcomes across the globe, while cultural variation and specific local interpretations may occur.

## 5. Towards an Inclusive Research Agenda

This integrative review explores the profound and multifaceted social–psychological consequences of the COVID-19 pandemic, demonstrating that psychological responses extended far beyond individual distress to encompass broader patterns of social cohesion, resilience, and community functioning. By bringing together theoretical frameworks and recent empirical contributions, the Special Issue “Mental Health Consequences of COVID-19: The Role of Social Determinants” underscores how the pandemic simultaneously exposed vulnerabilities and mobilized social resources across diverse populations. The studies reviewed here converge on the insight that social–psychological outcomes during health crises are shaped not merely by viral exposure or epidemiological risk but by social structures, identity processes, moral motivations, and community-level capacities for adaptation.

Across the studies, a consistent theme emerges: vulnerability is not evenly distributed. Gender disparities appear robust, as evidenced by both Hwang and Shin [20] and Domosławska-Żylińska and colleagues [21], who show that women disproportionately carried the psychological burden of the pandemic. These findings echo global evidence that caregiving responsibilities, economic precarity, and gendered social expectations magnified emotional exhaustion and mental health decline. Likewise, Grajek and colleagues [22] illustrate how pre-existing health vulnerabilities (such as cancer diagnosis and treatment) intensified pandemic-related anxiety, pointing to the need for clinically sensitive mental health interventions during public health emergencies. At the same time, the studies by Thomson and colleagues [23] and review by Wider and colleagues [24] remind us that not all responses to crises are negative. Engagement with culture, nature, and prosocial behavior can serve as powerful buffers, reinforcing social connectedness, emotional well-being, and community resilience. This duality reveals the importance of holistic approaches that integrate individual, relational, and societal factors.

The theoretical frameworks discussed earlier (i.e., social identity, moral psychology, and resilience theory) provide important conceptual anchors for interpreting these findings. Social identity processes help explain why some groups strengthened internal solidarity, while others experienced intergroup divisions or social fragmentation. Moral psychology clarifies why certain segments of the population responded with elevated cooperation, volunteerism, or adherence to health guidelines, whereas others reacted with moralized blame or resistance. Resilience frameworks offer a lens through which to understand community variability in coping, adaptation, and recovery. Indeed, transactional models of stress and coping [30] emphasize the role of cognitive appraisal, perceived controllability, and coping strategies in shaping psychological outcomes, again reflecting how such social–psychological consequences of major events are often a result (or rather a product) of individual and contextual factors [31,32]. Together, these perspectives suggest that psychological outcomes during collective crises depend as much on social meaning systems as they do on individual traits or demographic attributes.

### 5.1. From Closing the Gaps…

Despite the progress made, the reviewed literature collectively highlights several remaining gaps. First, most studies rely on cross-sectional designs, limiting causal inference about the emergence, persistence, and long-term consequences of pandemic-related distress or resilience. Second, many forms of structural inequality (including socioeconomic status, migration background, and access to technology) remain understudied in relation to social–psychological outcomes. For instance, the necessity of remote healthcare delivery has spurred growth in digital mental health services. Tanhan and colleagues [33] propose a framework emphasizing culturally adapted, accessible online platforms designed to sustain mental well-being during pandemics. Online interventions, such as teletherapy, psychoeducation, and peer support, have shown promise, particularly in reducing loneliness and anxiety, but face challenges related to digital literacy, privacy, and efficacy across diverse populations [34]. Integrating social–psychological principles, such as fostering community and moral engagement, can serve as a first step in enhancing these interventions’ impact.

Third, the variability across cultural contexts demonstrates the necessity of context-sensitive approaches during the development and rigorous evaluation of digital mental health tools, community-based interventions, and identity-based strategies. The role of responsible communication herein is paramount [35], as positive and accurate messaging can promote protective behaviors and resilience [36]. In that respect, I encourage future studies to target countries that have been under-represented in research (e.g., from the African continent) and integrate several life-threatening pandemics (e.g., HIV, Hepatitis C Virus, or other pre-existing health conditions) into a so-called syndemic approach [37]. This perspective recognizes that psychological vulnerability often emerges at the intersection of multiple, co-occurring health threats rather than from a single disease process. Hence, the mental health consequences observed among oncology patients [22] can be situated within a broader framework of compounded risk that also characterizes other populations experiencing layered stressors. Integrating COVID-19 with existing infectious disease burdens would allow us to identify shared and disease-specific mechanisms of vulnerability and resilience across contexts and better capture how intersecting epidemics, social inequalities, and health system capacities jointly shape mental health outcomes and intervention effectiveness.

### 5.2. …To Specific Policies

Taken together, theoretically grounded and evidence-based public health strategies should prioritize vulnerable groups (e.g., women, young adults, clinical populations, ethnic–cultural minority members, and socioeconomically disadvantaged communities) ensuring that support systems are both equitable and culturally responsive. Structural factors such as socioeconomic status, education, and access to healthcare must be prioritized in policy responses [38]. For vulnerable populations, access to mental healthcare should be expanded. This includes improving access to telehealth services, which have been shown to reduce anxiety and depression, particularly those for people with mobility issues or living in rural areas [39]. Policies should also target inequalities in these areas as they have direct impacts on mental health outcomes [40]. To strengthen community resilience even further, governments and public health organizations must foster trust [41]. This can be achieved through transparent communication and ensuring public health messages are inclusive and culturally sensitive [42]. In that sense, policies should encourage local leaders to be involved in the dissemination of public health information and community solidarity initiatives [43].

Finally, acknowledging that the psychological impacts of a pandemic extend beyond the crisis, governments should consider setting up long-term mental health monitoring systems. These would facilitate the early detection of emerging mental health problems and enable timely intervention [44,45]. These programs should encourage outdoor physical activity, such as nature walks, gardening, or outdoor fitness classes, as ways to reduce anxiety and depression [46]. Involving people in such resilience-building activities can empower them and foster stronger social bonds during future crises [47]. Moreover, outreach programs could entail support groups that address specific stressors such as caregiving and employment insecurity among women, uncertainty about academic progression and future employment among youngsters, reduced autonomy and digital exclusion among the elderly, and financial strain, housing and food insecurity among socioeconomically disadvantaged individuals.

To conclude, the COVID-19 pandemic has served as a global stress test for the psychological and social fabric of societies. This review demonstrates that the complex social–psychological consequences are neither uniform nor unidirectional: they reflect a complex interplay of adversity and adaptation, disconnection and solidarity, vulnerability and resilience. Integrating psychological science into public health policy is therefore essential for strengthening societies’ capacity not only to withstand future crises but to emerge from them more informed, cohesive, adaptive, and resilient.
